# Characteristics of head injuries in children at King Abdullah Specialized Children's Hospital, Jeddah: a cross-sectional study

**DOI:** 10.3389/fped.2026.1838275

**Published:** 2026-08-14

**Authors:** Sara Abed, Bashaer Gutah, Fatma Nasraldin, Ghaidaa Gadi, Muhammad Anwar Khan, Mansour Al Qurashi

**Affiliations:** 1 Pediatrics Department, King Abdullah Specialized Children’s Hospital, Ministry of National Guard Health Affairs, Jeddah, Saudi Arabia; 2 College of Medicine, King Saud Bin Abdulaziz University for Health Sciences, Jeddah, Saudi Arabia; 3 Research and Development, King Abdullah International Medical Research Center, Jeddah, Saudi Arabia; 4 Neonatology Department, King Abdullah Specialized Children’s Hospital, Ministry of National Guard Health Affairs, Jeddah, Saudi Arabia

**Keywords:** age stratification, length of stay, pediatric head trauma, Saudi Arabia, subgaleal hematoma, traumatic brain injury

## Abstract

**Introduction:**

Head injuries in children are a leading cause of death and long-term disability worldwide. However, detailed data on the characteristics of pediatric head injuries across different regions of Saudi Arabia remain limited. In this study, conducted at King Abdullah Specialized Children's Hospital (KASCH) in Jeddah, we aimed to identify the distinctive patterns of head injuries in children.

**Methods:**

This retrospective cross-sectional study included 308 pediatric patients aged <14 years who presented to KASCH between 2016 and 2023. Patients were categorized into three age groups (<1, 1–6, and >6–14 years). Data on demographics, injury types and causes, clinical presentation, imaging modalities, disabilities, Pediatric Glasgow Coma Scale scores, ophthalmologic findings, and hematological profiles were extracted from the BESTCare system. Comparisons across age groups were performed for injury mechanisms, types, and clinical presentation, and associations between injury type and length of hospital stay were evaluated.

**Results:**

Most patients were male (67.9%), with a mean age of 4.07 ± 3.9 years. Among patients aged <1 year, falls (76.3%), subgaleal hematoma (27.7%), and head swelling (43.5%) were the most common. In those aged 1–6 years, falls (63.8%), lacerations (74.7%), and bleeding (75%) predominated. In patients aged >6–14 years, motor vehicle accidents (42.7%), subgaleal hematoma (24.1%), and bleeding (22.8%) were the most frequent. Lacerations and concussions were associated with shorter hospital stays, whereas other injury types were associated with longer stays.

**Discussion:**

Age-stratified differences in injury characteristics underscore the importance of age-specific approaches to prevention, diagnosis, and management of pediatric head injuries.

## 1 Introduction

Head injuries are a significant cause of morbidity and mortality across all age groups worldwide (1). In children, they are a leading cause of death and long-term disability, affecting over 3 million children globally each year, with a reported incidence of approximately 50 cases per 100,000 children (2). Internationally, most pediatric head injuries result from falls and motor vehicle collisions, whereas non-accidental and sports-related injuries are less common (3). In Saudi Arabia, road traffic accidents are the most common cause of head injuries in children, followed by pedestrian injuries and falls. At the same time, violence is the least common cause in this age group (4).

A head injury refers to any damage to the skull, brain, or blood vessels in the head (5). These injuries can range from mild (e.g., cuts, bumps, or contusions) to moderate or severe, including internal bleeding, brain damage, skull fractures, deep cuts, open wounds, or combinations thereof (5). Head injuries can be classified in various ways, including by the area of the brain involved, as either focal or diffuse (6). Focal injuries are confined to a specific brain region and include contusions, intracranial hematomas, and skull fractures (7). By contrast, diffuse injuries involve multiple brain regions and include axonal injury, hypoxic–ischemic injury, diffuse cerebral edema, and microvascular injury (7). Although injuries may be categorized as predominantly focal or diffuse, most are heterogeneous, comprising both components (6). Another classification distinguishes between primary and secondary injuries. Primary brain injuries result directly from mechanical forces that disrupt brain tissue, whereas secondary injuries develop hours to days later due to indirect pathophysiological processes (6, 8).

The anatomy of a child's head differs from that of an adult. Pediatric skull bones are thinner, softer, and not fully calcified, providing less protection to the brain (9). Additionally, the developing brain has incomplete myelination, making it more susceptible to traumatic injury (10). In infants and young children, particularly those aged <5 years, head injuries often result from falls due to poor coordination and a relatively larger head size. As children grow older and become more independent, the causes shift toward higher-risk mechanisms, such as motor vehicle accidents (MVAs) (1).

There are notable differences between neurotrauma in pediatric and adult patients (11). Pediatric brain injury exhibits unique biomechanical properties, with external forces being absorbed differently owing to increased plasticity and deformability (12). These differences influence recovery and may increase vulnerability to long-term functional impairment, leading to poorer cognitive, behavioral, and developmental outcomes than in adults (13). Moreover, the evolving nature of the developing brain means that symptoms of traumatic brain injury may change over time, complicating diagnosis and management and potentially contributing to poorer outcomes (14).

A recent study conducted at King Saud Medical City (KSMC) in Riyadh, Saudi Arabia, provided valuable insights into pediatric head injuries, focusing on prehospital care and examining associations between injury causes, age groups, and injury severity using the Glasgow Coma Scale (GCS) and Injury Severity Score (ISS) (1). However, data from other regions in Saudi Arabia, particularly from specialized pediatric centers, remain limited, highlighting a regional gap. Furthermore, detailed characterization of head injury features, including symptoms and long-term disabilities, was limited in that study. To address this gap, we examine associations among injury causes, injury types, and symptoms across three age groups (<1, 1–6, and 6–14 years) and evaluate long-term disabilities. Accordingly, we aim to characterize pediatric head injuries at King Abdullah Specialized Children's Hospital (KASCH) in Jeddah, contributing to a broader understanding of pediatric head injuries in specialized pediatric healthcare settings in Saudi Arabia.

## 2 Methods

### 2.1 Study design and study settings

This retrospective cross-sectional study was performed at KASCH from 2016 to 2023. This design facilitates the analysis of pre-existing medical data without requiring additional data collection or patient follow-up, while remaining efficient and cost-effective. Participants were children from birth up to 14 years of age who presented to the emergency department and were admitted to an inpatient (IP) ward or the pediatric intensive care unit (PICU) or presented in an outpatient (OP) clinic with a head injury.

### 2.2 Participants

This study included all children admitted with head injury from birth up to 14 years of age. The inclusion of children up to 14 years reflects our hospital's age categorization, which ranges from birth to 14 years of age. Patients were divided into three age groups for comparison: <1 year, 1–6 years, and 6–14 years. Patients admitted with multiple trauma but no head injury other than superficial scalp cuts and abrasions, as well as those with pre-existing neurological disorders that could complicate assessment, were excluded.

### 2.3 Data source and variables

The data were collected using the BESTCare system through a non-probability consecutive sampling technique. BESTCare is a unified database used within the Ministry of National Guard Health Affairs (MNGHA) that contains patient medical records and supports a smart hospital approach, facilitating services for both patients and physicians (15).

The data collection sheet included demographic variables, such as serial number, date of birth, age at diagnosis, gender, weight (kg), height (cm), and body mass index (BMI). Injury-related variables included types of skull fractures (frontal, parietal, temporal, or occipital), traumatic vascular injuries (epidural hematoma, subdural hematoma, subarachnoid hematoma, subgaleal hematoma, or intraparenchymal hemorrhage), and other parenchymal injuries (cerebral contusion, cerebral laceration, cerebral edema, cerebral ischemia, axonal injury, or encephalomalacia). Some patients had multiple injury types.

Additional variables included trauma and admission details, such as patient type (OP, IP, or emergency patient), diagnosis date, imaging modalities (x-ray, computed tomography [CT], magnetic resonance imaging [MRI], or skeletal survey [SS]) based on clinical presentation, cause of injury (MVA, fall, hit, sport injury, violence, or unknown), presenting signs and symptoms (bleeding, seizure, dizziness, headache, head swelling, loss of consciousness [LOC], vomiting, amnesia, confusion, double vision, sleepiness, facial ecchymosis, periorbital ecchymosis, periorbital swelling, ear bleeding, epistaxis, retinal hemorrhage, none, or unknown), associated injuries (e.g., facial wound, facial nerve palsy, auditory canal injury, internal organ injury, spinal injury, limb fractures, chest bone fracture, facial bone fracture, or none), need for surgical intervention, new disability (e.g., bedridden status, memory loss, epilepsy, subglottic stenosis, ruptured eye, vocal cord paralysis, external nasal deformity, hearing problems, dysarthria, or behavioral changes), known perpetrator (father, mother, sibling, relative, friend, animal, unknown, or none), mortality status, Pediatric GCS (PGCS) score at presentation, indication for eye examination, and hematological profile. Multiple-choice responses were used for some variables (e.g., imaging modalities, symptoms, associated injuries, and disabilities). Clinical decision rules similar to the Pediatric Emergency Care Applied Research Network (PECARN) criteria are used in our emergency department to safely withhold CT imaging in low-risk children, thereby avoiding unnecessary radiation exposure. MRI is reserved for suspected diffuse axonal injury, non-accidental trauma evaluation, or secondary neurological deficits, although its use in the active setting is limited by the need for sedation. Children admitted or observed without advanced neuroimaging underwent short-term neurological observation, wound management, pain control, or suspected child abuse workup.

The PGCS is a neurological assessment tool used to evaluate the level of consciousness in children. It assesses eye opening (score: 1–4), verbal response (score: 1–5), and motor response (score: 1–6). Total scores are interpreted as follows: 3–8 (severe), 9–12 (moderate), and 13–15 (mild dysfunction) (16).

An SS is a series of whole-body x-rays used to identify occult fractures and confirm suspected child physical abuse (17). Eye examination by an ophthalmologist is performed in cases of suspected abusive head injury, particularly to assess for retinal hemorrhages (18). These evaluations help distinguish accidental from non-accidental (abusive) head injuries. Some patients with suspected abuse may require referral to the Suspected Child Abuse and Neglect (SCAN) team, social services, or child protection services.

Hematological profiles (e.g., factor VII deficiency, elevated prothrombin time [PT], elevated partial thromboplastin time [PTT], sickle cell anemia, or unspecified coagulation disorders) were recorded because these conditions may increase the risk of bleeding or ischemia following head injury.

### 2.4 Handling of undocumented data

Hospital length-of-stay data were missing for 18 of 308 patients. The median value (10 days) was used to impute missing values, allowing inclusion of all patients in the analysis, particularly as length of stay was a primary comparison variable. Stays exceeding the median (>10 days) were defined as prolonged and analyzed using logistic regression to assess associations with injury type.

### 2.5 Study outcomes

We compared three age groups (<1 year, 1–6 years, and 6–14 years) with respect to the cause and type of injury, and the four most common symptoms, to characterize primary head injury patterns within each age group. Additionally, the relationship between common injury types and hospital length of stay, including emergency room (ER) and PICU stays, was evaluated to reflect injury severity. Logistic regression was used to assess the association between injury type and prolonged hospital stay. Because this was a retrospective chart review, PGCS scores were available only when documented in the medical records. Undocumented PGCS values were recorded as missing and not imputed. To evaluate the potential impact of missing PGCS data, we compared the characteristics of patients with documented and undocumented PGCS scores (Supplementary Table S1).

### 2.6 Ethical consideration

The Institutional Review Board (IRB) of King Abdullah International Medical Research Center, Saudi Arabia, approved access to patient medical records (IRB approval number: IRB/0704/24). Informed consent was not required because the study was a retrospective chart review.

### 2.7 Data analysis

Continuous variables were presented as means and standard deviations, and categorical variables were summarized using percentages. Additionally, the Chi-square test and Fisher's exact test were used to analyze the categorical data. The Mann–Whitney test was used to evaluate the relationship between a continuous variable and a categorical variable. A *p*-value <0.05 was considered statistically significant. All statistical analyses were performed using SPSS version 20.0 (IBM Corp., Armonk, NY, USA).

## 3 Results

### 3.1 Demographic characteristics

In this study, all 947 patients admitted to the hospital between 2016 and 2023 were screened; however, only 308 met the inclusion criteria. Among the included patients, 209 (67.9%) were male, and 99 (32.1%) were female. A total of 59 patients (19.2%) were aged <1 year, 174 (56.5%) were aged 1–6 years, and 75 (24.4%) were aged >6–14 years. The mean age at diagnosis was 4.07 ± 3.9 years. Patients with head trauma were recruited from the emergency department (219, 71.1%), IP wards (72, 23.4%), and OP clinics (17, 5.5%). Most cases were accidental (305, 99.03%), while only three patients (0.97%) were confirmed abuse cases.

Following assessment, the GCS score was recorded in 116 patients (37.7%), with a mean score of 13.79 ± 2.73 points (range: 3–15). A comparison of patients with documented and undocumented PGCS scores revealed significant differences in sex (*p* = 0.019), age (*p* < 0.001), ICU admission (*p* = 0.028), and mechanism of injury (*p* = 0.033) (Supplementary Table S1). The length of stay in the hospital and ER was documented for 290 patients (94.2%), with a mean of 3.11 ± 8.43 days (range: 1–107). The length of stay in the PICU was recorded for 26 patients (8.4%), with a mean of 5.19 ± 5 days (range: 1–25). BMI was available for 186 patients (60.4%), with a mean of 15.95 ± 3.69 kg/m^2^ (range: 9.93–44.36 kg/m^2^).

### 3.2 Injury types and causes

Injuries were assessed radiologically using CT scans in 180 patients (58.4%), MRI in 21 (7%), x-rays in 16 (5.5%), and SSs in 11 (3.6%). Eye examinations were performed in 13 patients (4.2%). Hematological abnormalities included factor VII deficiency, elevated PT, elevated PTT, and sickle cell anemia, each identified in one patient (0.3%), while two patients (0.6%) had unspecified coagulation disorders. Referrals included the SCAN team for four patients (1.3%) and social services for two patients (0.6%).

The perpetrators were identified in 22 cases (7.1%), most commonly siblings (12 cases, 3.9%), followed by mothers (3 cases, 1%), relatives (2 cases, 0.6%), friends (2 cases, 0.6%), animals (2 cases, 0.6%), and fathers (1 case, 0.3%).

**Figure 1:**
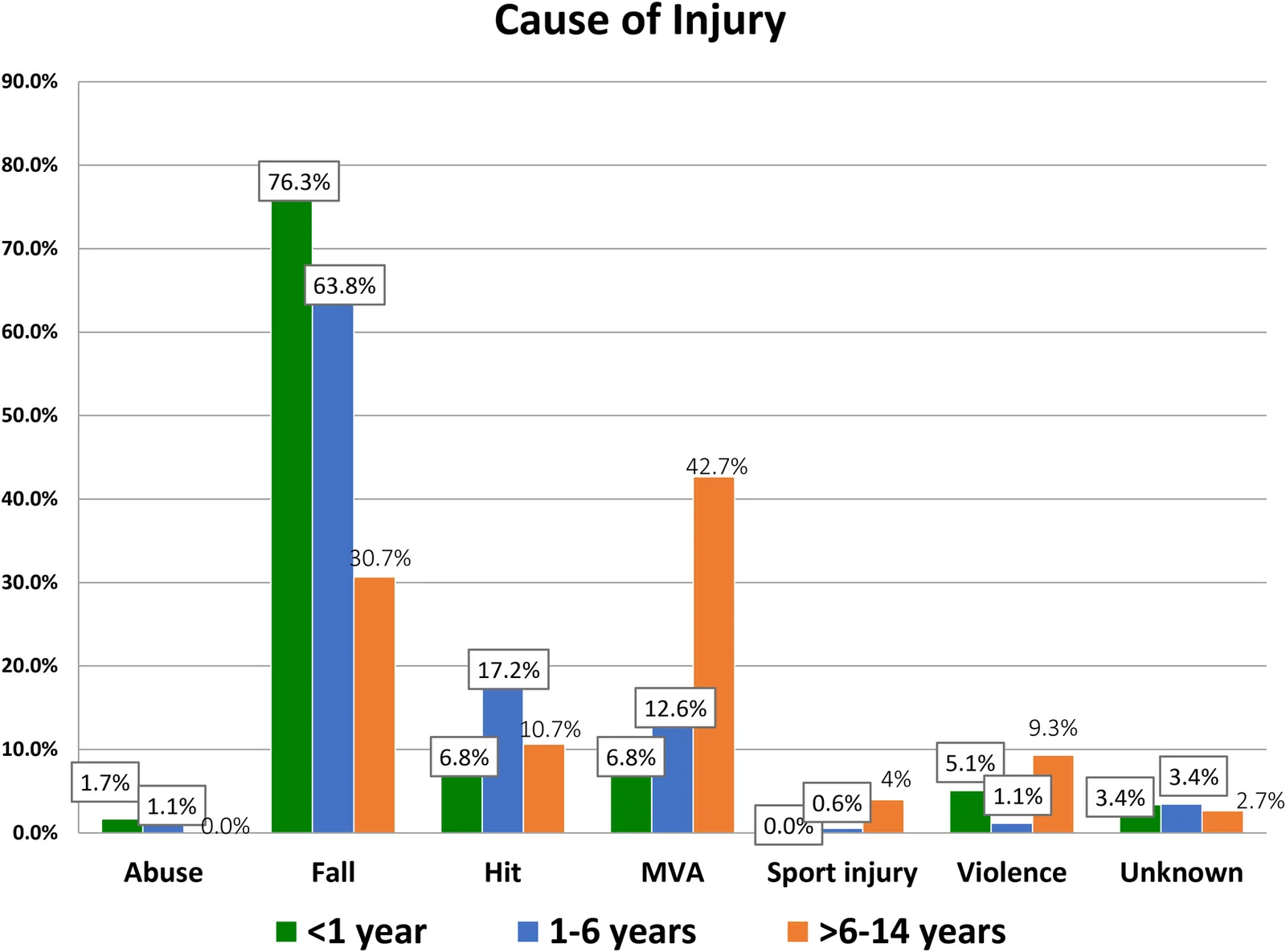
Causes of injury according to age group.

The most common cause of injury overall was falls (179 patients, 58.1%), followed by MVAs (58 patients, 18.8%), hits (42 patients, 13.6%), violence (12 patients, 3.9%), unknown causes (10 patients, 3.2%), and sports injuries (4 patients, 1.3%). Abuse was the least common cause (three patients, 1%). Falls were the leading mechanism in patients aged <1 year (45 patients, 76.3%) and 1–6 years (111 patients, 63.8%), whereas MVAs were most common in patients aged >6–14 years (32 patients, 42.7%). The second most common causes varied by age group: among patients <1 year, MVAs and hits (four patients each, 6.8%); among patients aged 1–6 years, hits (30 patients, 17.2%); and among patients aged >6–14 years, falls (23 patients, 30.7%) (Figure 1).

Head injury types varied, with subgaleal hematoma being the most common (141 patients, 45.8%), followed by skull fractures (102 patients, 33.1%). The most frequently fractured bones were the parietal (53 patients, 17.2%), frontal (29 patients, 9.4%), occipital (25 patients, 8.1%), and temporal (21 patients, 6.8%) bones. Lacerations were identified in 99 patients (32.1%), followed by subdural hematoma (31 patients, 10.1%), cerebral concussion (19 patients, 6.2%), epidural hematoma (11 patients, 3.6%), subarachnoid hematoma (9 patients, 2.9%), and intraparenchymal hemorrhage (6 patients, 1.9%).

Other less common head injuries were observed in 18 patients: axonal injury (9 patients, 50%), contusion (5 patients, 27.8%), cerebral edema (2 patients, 11.1%), cerebral ischemia (1 patient, 5.6%), and encephalomalacia (1 patient, 5.6%).

### 3.3 Clinical characteristics

Patients presented with a range of symptoms. The most common were bleeding from the injury site (92 patients, 29.9%), vomiting (75 patients, 24.4%), head swelling (46 patients, 14.9%), LOC (43 patients, 14%), seizures or abnormal movements (27 patients, 8.8%), headache (26 patients, 8.4%), and dizziness (24 patients, 7.8%).

Less common symptoms were grouped into four categories. The first included facial signs (19 patients): periorbital ecchymosis (10 patients, 52.6%), facial ecchymosis (5 patients, 26.3%), and periorbital swelling (4 patients, 21.1%). The second included bleeding at other sites (17 patients): epistaxis (9 patients, 52.9%) and ear bleeding (8 patients, 47.1%). The third included other neurological symptoms (17 patients): sleepiness (8 patients, 47.1%), confusion (5 patients, 29.4%), amnesia (2 patients, 11.8%), and double vision (2 patients, 11.8%). The fourth included other associated injuries (10 patients): auditory canal injury (4 patients, 40%), facial nerve palsy (4 patients, 40%), and facial wounds (2 patients, 20%).

Severe associated injuries included facial bone fractures (20 patients, 6.5%), lower limb fractures (13 patients, 4.2%), chest bone fractures (10 patients, 3.2%), spinal cord injury (9 patients, 2.9%), upper limb fractures (8 patients, 2.6%), and internal organ injuries (6 patients, 2%), including liver and lung injuries (3 patients each, 1%). Retinal hemorrhage was observed in two patients (0.6%), associated with MVA and abuse.

A total of 10 new disabilities were identified in 10 patients. The most common was epilepsy (three patients, 0.97%), followed by memory loss (two patients, 0.6%). Other disabilities (each in one patient, 0.3%) included bedridden status, subglottic stenosis, ruptured eye, vocal cord paralysis, external nasal deformity, hearing problems, dysarthria, and behavioral changes. Some patients had multiple disabilities. Death was documented in one patient (0.3%).

### 3.4 Age group comparison

Overall, the most common head injuries were subgaleal hematoma, skull fracture, laceration, and subdural hematoma. However, variations were observed across age groups. In patients aged <1 year and >6–14 years, subgaleal hematoma was most common (39 patients [27.7%] and 34 patients [24.1%], respectively), followed by skull fracture (32 patients [31.4%] and 24 patients [23.5%], respectively). Among patients aged 1–6 years, laceration was the most common injury (74 patients, 74.7%), followed by subgaleal hematoma (68 patients, 48.2%).

The least common injuries were epidural hematoma in patients <1 year (one patient), intraparenchymal hemorrhage in patients aged 1–6 years (one patient), and subarachnoid hematoma in patients aged >6–14 years (three patients). Injury types with *p* < 0.05 were considered age-related, whereas cerebral concussion, epidural hematoma, and other less common injuries were not age-related (Table 1).

**Table 1:** Types of injury according to age groups.

Type of injury	Types of injury compared to age groups						*p*
	<1 year		1–6 years		>6–14 years		
	*n*	(%)	*n*	(%)	*n*	(%)	
Skull fracture	32	(31.4)	46	(45.1)	24	(23.5)	<0.001
Laceration	2	(2.0)	74	(74.7)	23	(23.2)	<0.001
Subgaleal hematoma	39	(27.7)	68	(48.2)	34	(24.1)	0.002
Subdural hematoma	10	(32.3)	11	(35.5)	10	(32.3)	0.036
Subarachnoid hematoma	4	(44.4)	2	(22.2)	3	(33.3)	0.046
Epidural hematoma	1	(9.1)	5	(45.5)	5	(45.5)	0.307
Intraparenchymal Hemorrhage	0	(0.0)	1	(16.7)	5	(83.3)	0.010
Cerebral concussion	0	(0.0)	14	(73.7)	5	(26.3)	0.053
Other injuries							
Axonal injury	1	(11.1)	2	(22.2)	6	(66.7)	0.569
Cerebral edema	0	(0.0)	1	(50.0)	1	(50.0)	
Cerebral ischemia	0	(0.0)	1	(100.0)	0	(0.0)	
Contusion	1	(20.0)	3	(60.0)	1	(20.0)	
Encephalomalacia	0	(0.0)	1	(100.0)	0	(0.0)	

Overall, the most common symptoms were bleeding, vomiting, head swelling, and LOC. However, their distribution varied by age group. In patients aged <1 year, head swelling was the most common (20 patients, 43.5%), whereas bleeding was the most common in patients aged 1–6 years (69 patients, 75%) and in those aged 6–14 years (21 patients, 22.8%). The least common symptom in patients <1 year was bleeding (two patients, 2.2%), while in the older groups, it was head swelling (21 patients [45.7%] and 5 patients [10.9%], respectively). Only bleeding and head swelling showed significant differences among age groups (*p* < 0.05) (Table 2).

**Table 2:** Symptoms according to age groups.

Symptom	Symptoms compared to age groups						*p*
	<1 year		1–6 years		>6–14 years		
	*n*	(%)	*n*	(%)	*n*	(%)	
Bleeding	2	(2.2)	69	(75.0)	21	(22.8)	<0.001
Vomiting	14	(18.7)	47	(62.7)	14	(18.7)	0.369
Head swelling	20	(43.5)	21	(45.7)	5	(10.9)	<0.001
LOC	5	(11.6)	24	(55.8)	14	(32.6)	0.239

LOC, loss of consciousness.

### 3.5 Duration of stay

The length of hospital and ER stay was compared across injury types. Patients with skull fractures (including frontal, occipital, parietal, and temporal fractures) had significantly longer hospital stays than those without fractures (*p* < 0.05). Conversely, patients with lacerations had significantly shorter hospital stays (*p* < 0.001) (Table 3).

**Table 3:** Hospital length of stay (including emergency room [ER]) according to injury types.

Type of injury	No/Yes	*N*	Mean rank	Median	*p*
Skull fracture					
Length of stay (incl. ER)	No	206	133.9	1.0	<0.001
	Yes	102	196.0	2.0	
Frontal bone fracture					
Length of stay (incl. ER)	No	279	149.2	1.0	<0.001
	Yes	29	205.6	2.0	
Occipital bone fracture					
Length of stay (incl. ER)	No	283	150.0	1.0	0.001
	Yes	25	205.5	2.0	
Parietal bone fracture					
Length of stay (incl. ER)	No	255	144.5	1.0	<0.001
	Yes	53	202.7	2.0	
Temporal bone fracture					
Length of stay (incl. ER)	No	287	150.2	1.0	0.001
	Yes	21	213.6	3.0	
Laceration					
Length of stay (incl. ER)	No	209	169.0	2.0	<0.001
	Yes	99	123.9	1.0	
Subgaleal hematoma					
Length of stay (incl. ER)	No	167	144.6	1.0	0.021
	Yes	141	166.2	2.0	
Subdural hematoma					
Length of stay (incl. ER)	No	277	149.0	1.0	<0.001
	Yes	31	203.5	2.0	
Subarachnoid hematoma					
Length of stay (incl. ER)	No	299	152.6	1.0	0.018
	Yes	9	217.7	3.0	
Epidural hematoma					
Length of stay (incl. ER)	No	297	152.5	1.0	0.022
	Yes	11	209.8	2.0	
Intraparenchymal hemorrhage					
Length of stay (incl. ER)	No	302	152.4	1.0	0.001
	Yes	6	259.6	7.5	
Cerebral concussion					
Length of stay (incl. ER)	No	289	157.1	2.0	0.031
	Yes	19	115.4	1.0	

Similarly, patients with subgaleal hematoma, subdural hematoma, subarachnoid hematoma, epidural hematoma, and intraparenchymal hemorrhage had significantly longer hospital stays than those without these conditions (*p* < 0.05). By contrast, patients with cerebral concussion had significantly shorter hospital stays (*p* < 0.05) (Table 3). No significant differences were observed in PICU length of stay across injury types (*p* > 0.05) (Table 4).

**Table 4:** Pediatric intensive care unit (PICU) length of stay according to injury types.

Type of Injury	Length of stay (PICU)		
	Mean	(SD)	*p*
Skull fracture	5.5	(5.7)	0.705
Frontal bone fracture	5.6	(3.3)	0.820
Occipital bone fracture	4.6	(2.6)	0.775
Parietal bone fracture	5.8	(7.2)	0.634
Temporal bone fracture	7.3	(8.3)	0.403
Laceration	1.5	(0.7)	0.286
Subgaleal hematoma	6.1	(5.9)	0.236
Subdural hematoma	4.6	(2.6)	0.708
Subarachnoid hematoma	3.0	—	0.664
Epidural hematoma	2.0	—	0.526
Intraparenchymal hemorrhage	6.0	(3.6)	0.773
Cerebral concussion	—	—	—

SD, standard deviation.

Additionally, multivariate logistic regression was performed to identify predictors of prolonged hospital stay. Length of stay >10 days was coded as the outcome variable. The overall model was significant (Omnibus test, *p* < 0.05), with a Nagelkerke *R*^2^ of 0.387, indicating that the predictors explained 38.7% of the variance. Among the independent variables, only occipital bone fracture and subdural hematoma were significant predictors, whereas other variables were not (Table 5).

**Table 5:** Multivariable logistic regression analysis of predictors of prolonged hospital stay.

Variable	Category	OR	95% CI	*p*
Age when diagnosed		1.105	(1.11–0.80)	0.543
Age diagnosis	<1 year	1		
	1–6 years	.762	(.05–11.63)	0.745
	>6–14 years	3.86	(.06–246.08)	0.524
Skull fracture	No	1		0.962
	Yes	.945	(.09–9.82)	
Frontal bone fracture	No	1		0.986
	Yes	1.02	(.12–8.58)	
Occipital bone fracture	No	1		0.047
	Yes	7.42	(1.03–53.59)	
Parietal bone fracture	No	1		0.495
	Yes	1.93	(.29–12.77)	
Temporal bone fracture	No	1		0.179
	Yes	4.11	(.52–32.35)	
Subgaleal hematoma	No	1		0.423
	Yes	1.83	(.42–7.95)	
Subdural hematoma	No	1		0.029
	Yes	6.24	(1.21–32.24)	
Subarachnoid hematoma	No	1		0.999
	Yes	.000	(0.00)	
Epidural hematoma	No	1		0.999
	Yes	.000	(0.00)	
Constant		6.471		<0.001

## 4 Discussion

### 4.1 Overview of the main findings

Our study provides insight into the clinical characteristics of pediatric head trauma in a tertiary hospital in Saudi Arabia. The findings indicate a young mean age of approximately 4 years, with a predominance of male patients. Falls were identified as the most common mechanism of injury, particularly among younger children. Subgaleal hematoma, skull fractures, and scalp lacerations were the most frequently observed injuries. Clinical presentation and severity varied across age groups, with generally favorable outcomes and a low mortality rate.

A recent study using data from the Saudi Trauma Registry (STAR) at KSMC included 466 children with traumatic head injuries from 2017 to 2022 (1). Although both studies address similar topics, several differences were noted. The present study provides more detailed classifications of injury types, including specific hematoma types and fractured skull bones. Additionally, the inclusion of secondary injuries, associated disabilities, and referral patterns, such as cases referred to the SCAN team, adds an important dimension. By contrast, the STAR study emphasized injury severity scores (GCS, ISS) and prehospital care details, which were not consistently available in our database (1). These differences likely reflect variations in database structure, study objectives, and available clinical data.

### 4.2 Demographic characteristics

The present study shows a mean age at diagnosis of 4.07 years. In comparison, a study conducted in Riyadh involving 1,219 pediatric patients with traumatic head injuries reported a mean age of 8.9 years (4). Male individuals constituted the majority of cases in both studies, consistent with the male predominance observed here. This may be attributed to behavioral and social factors, as male children are often more engaged in physically active or risk-prone activities, increasing their risk of traumatic head injuries.

### 4.3 Mechanisms of injury

Falls were identified as the leading mechanism of injury in the current study, particularly among children aged <6 years. By contrast, the Riyadh study reported MVAs as the most common cause (4). Differences in study populations may explain this discrepancy. The present study included a higher proportion of younger children, who are more prone to falls due to developmental factors and increased activity levels. Conversely, the Riyadh study included older children and adolescents, who are more exposed to traffic-related risks. Additionally, temporal differences between the studies (2001–2009 vs. 2016–2023) may reflect improvements in road safety policies. Our findings were consistent with those of Hana et al., who reported that simple trips and falls were the predominant mechanisms of pediatric head trauma, with injury mechanisms varying according to age and developmental stage (22), highlighting the importance of considering age-specific injury mechanisms when evaluating pediatric head trauma.

### 4.4 Injury patterns and clinical characteristics

The injury patterns observed in this study are consistent with those previously reported. Subgaleal hematoma was the most frequent injury type, followed by skull fractures and scalp lacerations. The prevalence of skull fractures and intracranial injuries aligns with that in a national overview in the United Kingdom, which reported that approximately 10% of children with head injuries had either an intracranial injury or a skull fracture (19). The high prevalence of subgaleal hematoma may be attributed to the fragility of scalp vessels and anatomical characteristics in pediatric patients.

Injury patterns and clinical presentations also varied across age groups. Infants most commonly present with head swelling, reflecting the higher incidence of subgaleal hematomas and anatomical features of the infant skull. By contrast, bleeding from the injury site was more frequently observed among older children, likely owing to a higher incidence of lacerations and exposure to more forceful injury mechanisms. These findings underscore the importance of age-specific clinical assessment in pediatric head trauma. Similarly, Hana et al. reported that anatomical injury patterns differ by age, with younger children demonstrating greater susceptibility to focal head injuries, whereas injury distribution becomes more diffuse with increasing age. These findings further support the influence of developmental anatomy and age-related mechanisms on injury patterns (20). Associated systemic injuries, including facial bone fractures, limb fractures, spinal injuries, chest bone fractures, and internal organ injuries, were observed in our cohort. However, the long-term prognosis of these injuries was not specifically evaluated owing to the study's retrospective design and limited follow-up data.

Diagnostic imaging primarily relied on CT, which remains the standard modality for evaluating pediatric head trauma owing to its ability to detect skull fractures and intracranial hemorrhage rapidly (21). MRI is typically reserved for detailed evaluation of subtle brain injuries such as microbleeds and axonal injuries (22). Hana et al. emphasized the importance of mechanism-based clinical assessment and selective diagnostic imaging in pediatric head trauma, reporting that larger scalp hematomas were associated with imaging abnormalities, while vomiting alone was not predictive of abnormal imaging findings. These observations reinforce the need to integrate clinical examination with injury mechanism when evaluating pediatric head injuries (20). Consistent with this approach, some patients in our cohort were managed based on clinical assessment alone and did not require neuroimaging.

### 4.5 Overall outcomes

Overall outcomes in this study were favorable. Mortality was very low, with only one recorded death (0.3%), which is substantially lower than the rates reported in other studies. For example, the Riyadh study reported a mortality rate of 20% among high school students with traumatic head injuries (4). In comparison, the KSMC study reported an overall mortality rate of 7.7%, with the highest rate (10.8%) observed among children aged 6–12 years (1).

Additionally, only a small proportion of patients required surgical intervention or developed new disabilities. These findings are consistent with those in global studies that highlight increasing variability in outcomes with increasing injury severity (19). The favorable outcomes observed in this study may reflect differences in injury severity, patient demographics, and access to timely medical care.

### 4.6 Predictors of hospital stay

An important finding of this study was the identification of predictors associated with prolonged hospital stay. In the initial comparisons, most types of skull fractures and hematomas documented were associated with longer hospitalization, likely reflecting more severe intracranial injury requiring closer monitoring. By contrast, lacerations and concussions were significantly associated with shorter hospital stays, possibly owing to their lower severity and the need for primarily symptomatic management without surgical intervention in most cases.

However, in the multivariate logistic regression analysis, only occipital bone fracture and subdural hematoma remained significant predictors of prolonged hospital stay, while other variables were not significantly associated with length of stay. Patients with occipital bone fractures had approximately sevenfold higher odds of prolonged hospitalization than those without such fractures (odds ratio [OR], 7.42). This is explained by the anatomical location of the occipital bone, which primarily forms the posterior cranial fossa and part of the skull base. It is also adjacent to the major dural venous sinuses (23). Therefore, occipital bone fracture may result in intracranial hematoma/hemorrhage, cerebrospinal fluid (CSF) leak, or cranial nerve palsies when the base of the skull is involved. As a result, patients would require assessment of these complications, which will prolong their hospital stay (24). Similarly, the presence of a subdural hematoma increased the odds of prolonged hospital stay by approximately sixfold (OR, 6.24). Although intraparenchymal hemorrhage and axonal injury are considered severe traumatic injuries, they did not remain significant in the multivariate analysis owing to the small number of affected patients in the study population. Subdural hematoma was the only hemorrhagic injury that remained an independent predictor after adjustment for injury types. This is associated with the higher number of affected patients and their frequent need for neurological monitoring, neuroimaging, and sometimes neurosurgery, thereby increasing patients' hospital stay (25). The findings suggest that these injuries may reflect more severe intracranial trauma requiring extended monitoring and care.

### 4.7 Strengths and limitations

The current study provides valuable insights into pediatric head injury patterns within a Saudi Arabian tertiary hospital setting. A key strength is the comprehensive stratification of injury mechanisms and types across pediatric age groups, which revealed significant associations between specific injuries and age categories. Furthermore, by executing an adjusted multivariate logistic regression model, we successfully isolated specific structural predictors of high-acuity outcomes, establishing that occipital bone fractures (OR = 7.42; *p* = 0.047) and subdural hematomas (OR = 6.24; *p* = 0.029) serve as powerful, independent drivers of a prolonged hospital stay exceeding 10 days. Additionally, the tracking of associated systemic injuries, secondary complications, and acute functional outcomes, such as new disabilities, offers a broader, more comprehensive clinical perspective on pediatric neurotrauma in the region.

Nevertheless, some limitations should be acknowledged. First, documented clinical parameters exhibited missingness within the electronic medical records. Crucially, our secondary sub-analysis revealed that the missing GCS data were not missing completely at random (Supplementary Table S1). Instead, a distinct selection and documentation bias was observed: clinicians significantly prioritized numeric GCS calculations for high-acuity presentations, such as MVAs (56.9%) and cases requiring intensive care unit admission (57.7%), whereas minor trauma or presentations in infants under 1 year of age (86.4% missingness) routinely defaulted to qualitative narrative tracking. This non-random missingness introduces documentation and attrition bias, limiting our capacity to generalize formal severity-stratified outcomes across the lowest-acuity tier of the cohort. Second, fall height could not be reliably assessed as a predictor because it was not documented for most patients. Furthermore, because of the retrospective, cross-sectional design based on acute clinical records, delayed neurodevelopmental, cognitive, or behavioral sequelae could not be reliably captured. Consequently, the true frequency of long-term disability is likely underestimated, as subtle functional deficits frequently remain latent during acute hospitalization and only manifest as the child encounters future developmental or academic milestones. Finally, as a single-center, hospital-based study conducted at a specialized children's facility, the results may reflect institutional triage and imaging protocols, meaning they may not fully represent the broader pediatric population, especially milder cases managed in community or non-specialized settings. Future multicenter studies with larger sample sizes and standardized, longitudinal tracking protocols are needed to improve generalizability and provide a more comprehensive understanding of pediatric head trauma across Saudi Arabia.

## 5 Conclusion

We investigated pediatric head injuries at KASCH in Jeddah from 2016 to 2023, providing a detailed characterization of injury mechanisms, clinical presentations, and acute outcomes within a specialized tertiary care setting. Most of the cohort comprised male patients and children aged 1–6 years. Low-energy falls and localized head swelling predominated among younger children, whereas high-velocity MVAs and active bleeding were the most common findings in older pediatric cohorts. Across all age groups, subgaleal hematomas, skull fractures, and lacerations constituted the most frequently observed injury types.

Using an adjusted multivariate logistic regression model, we address a critical regional gap by identifying independent structural predictors of prolonged hospitalization. Specifically, occipital bone fractures and subdural hematomas were established as powerful, independent drivers of a prolonged hospital stay exceeding 10 days, highlighting the necessity of conservative IP monitoring protocols for these specific craniocerebral lesions. Furthermore, a secondary evaluation of clinical parameters revealed a distinct triage selection bias, where formal numeric severity tracking via the GCS was selectively prioritized for higher-acuity presentations such as MVAs and intensive care unit admissions.

Our findings underscore distinct, age-stratified injury patterns and emphasize the importance of tailored diagnostic, management, and documentation approaches in pediatric emergency departments. While these data optimize our understanding of acute-phase pediatric neurotrauma, further multicenter, prospective research incorporating standardized documentation and longitudinal post-discharge protocols is warranted. Such efforts are essential to capture delayed neurodevelopmental sequelae fully and design targeted public health prevention strategies aimed at reducing the broader burden of pediatric head injuries in Saudi Arabia.

## Data Availability

The raw data supporting the conclusions of this article will be made available by the authors upon reasonable request, without undue reservation.
