# Supplementary material for: Characteristics of head injuries in children at King Abdullah Specialized Children's Hospital, Jeddah: a cross-sectional study
Source: Front Pediatr. 2026 Aug 14;14:1838275. doi: 10.3389/fped.2026.1838275 (PMC13522143; doi:10.3389/fped.2026.1838275)
Supplement: Supplementary file 1 [file Supplementaryfile1.docx]

Supplementary Material

# A comparison of patients with documented and undocumented PGCS scores, Supplementary Table S1

|  |  |  | **GCS Reporting** | | | |  |
| --- | --- | --- | --- | --- | --- | --- | --- |
|  |  | **Total** | **No** | | **Yes** | | **p** |
|  |  |  | **n** | **%** | **n** | **%** |  |
| **Gender** | |  |  |  |  |  |  |
|  | Male | 209 | 121 | (57.9) | 88 | (42.1) | 0.019* |
|  | Female | 99 | 71 | (71.7) | 28 | (28.3) |  |
| **Age** | |  |  |  |  |  |  |
|  | Birth to 5 years | 71 | 54 | (76.1) | 17 | (23.9) | 0.006* |
|  | 6–11 years | 161 | 101 | (62.7) | 60 | (37.3) |  |
|  | 12–15 years | 43 | 19 | (44.2) | 24 | (55.8) |  |
|  | ≥16 years | 33 | 18 | (54.5) | 15 | (45.5) |  |
| **Patient type** | |  |  |  |  |  |  |
|  | EP | 219 | 136 | (62.1) | 83 | (37.9) | 0.183* |
|  | IP | 72 | 42 | (58.3) | 30 | (41.7) |  |
|  | OP | 17 | 14 | (82.4) | 3 | (17.6) |  |
| **LOS_ER_10Median** | | |  |  |  |  |  |
|  | 10 days or less | 295 | 186 | (63.1) | 109 | (36.9) | 0.219* |
|  | >10 days (prolonged) | 13 | 6 | (46.2) | 7 | (53.8) |  |
| **ICU admission** | | |  |  |  |  |  |
|  | No | 282 | 181 | (64.2) | 101 | (35.8) | 0.028* |
|  | Yes | 26 | 11 | (42.3) | 15 | (57.7) |  |
| **Internal diagnosis** | | |  |  |  |  |  |
|  | Child abuse | 2 | 0 | (0.0) | 2 | (100.0) | 0.141† |
|  | Head trauma | 306 | 192 | (62.7) | 114 | (37.3) |  |
| **Age** | |  |  |  |  |  |  |
|  | <1 year | 59 | 51 | (86.4) | 8 | (13.6) | <0.001* |
|  | 1 to 6 years | 175 | 104 | (59.4) | 71 | (40.6) |  |
|  | >6 to 14 years | 74 | 37 | (50.0) | 37 | (50.0) |  |
| **Occipital bone fracture** | | |  |  |  |  |  |
|  | No | 283 | 176 | (62.2) | 107 | (37.8) | 0.858* |
|  | Yes | 25 | 16 | (64.0) | 9 | (36.0) |  |
| **Parietal bone fracture** | | |  |  |  |  |  |
|  | No | 255 | 158 | (62.0) | 97 | (38.0) | 0.765* |
|  | Yes | 53 | 34 | (64.2) | 19 | (35.8) |  |
| **Temporal bone fracture** | | | |  |  |  |  |
|  | No | 287 | 181 | (63.1) | 106 | (36.9) | 0.329* |
|  | Yes | 21 | 11 | (52.4) | 10 | (47.6) |  |
| **Laceration** | |  |  |  |  |  |  |
|  | No | 209 | 130 | (62.2) | 79 | (37.8) | 0.943* |
|  | Yes | 99 | 62 | (62.6) | 37 | (37.4) |  |
| **Subgaleal hematoma** | | |  |  |  |  |  |
|  | No | 167 | 111 | (66.5) | 56 | (33.5) | 0.104* |
|  | Yes | 141 | 81 | (57.4) | 60 | (42.6) |  |
| **Subdural hematoma** | | |  |  |  |  |  |
|  | No | 277 | 173 | (62.5) | 104 | (37.5) | 0.899* |
|  | Yes | 31 | 19 | (61.3) | 12 | (38.7) |  |
| **Subarachnoid hematoma** | | | |  |  |  |  |
|  | No | 299 | 188 | (62.9) | 111 | (37.1) | 0.261* |
|  | Yes | 9 | 4 | (44.4) | 5 | (55.6) |  |
| **Epidural hematoma** | | |  |  |  |  |  |
|  | No | 297 | 184 | (62.0) | 113 | (38.0) | 0.469* |
|  | Yes | 11 | 8 | (72.7) | 3 | (27.3) |  |
| **Frontal bone fracture** | | |  |  |  |  |  |
|  | No | 279 | 175 | (62.7) | 104 | (37.3) | 0.664* |
|  | Yes | 29 | 17 | (58.6) | 12 | (41.4) |  |
| **Skull fracture** | |  |  |  |  |  |  |
|  | No | 206 | 129 | (62.6) | 77 | (37.4) | 0.884* |
|  | Yes | 102 | 63 | (61.8) | 39 | (38.2) |  |
| **Intraparenchymal hemorrhage** | | | |  |  |  |  |
|  | No | 302 | 189 | (62.6) | 113 | (37.4) | 0.676† |
|  | Yes | 6 | 3 | (50.0) | 3 | (50.0) |  |
| **Cerebral concussion** | | |  |  |  |  |  |
|  | No | 289 | 179 | (61.9) | 110 | (38.1) | 0.572* |
|  | Yes | 19 | 13 | (68.4) | 6 | (31.6) |  |
| **Other injuries** | |  |  |  |  |  |  |
|  | Axonal injury | 9 | 2 | (22.2) | 7 | (77.8) | 0.153† |
|  | Cerebral edema | 2 | 0 | (0.0) | 2 | (100.0) |  |
|  | Cerebral ischemia | 1 | 1 | (100.0) | 0 | (0.0) |  |
|  | Contusion | 5 | 3 | (60.0) | 2 | (40.0) |  |
|  | Encephalomalacia | 1 | 1 | (100.0) | 0 | (0.0) |  |
| **Cause of injury** | | |  |  |  |  |  |
|  | Fall | 179 | 121 | (67.6) | 58 | (32.4) | 0.033* |
|  | Hit | 42 | 28 | (66.7) | 14 | (33.3) |  |
|  | MVA | 58 | 25 | (43.1) | 33 | (56.9) |  |
|  | Sport injury | 4 | 2 | (50.0) | 2 | (50.0) |  |
|  | Violence | 15 | 9 | (60.0) | 6 | (40.0) |  |
|  | Unknown | 10 | 7 | (70.0) | 3 | (30.0) |  |

GCS, Glasgow Coma Scale; EP, emergency patient; IP, inpatient; OP, outpatient; ICU, intensive care unit; MVA, motor vehicle accident; LOS: Length of Stay.
